# Secondary data in diabetes surveillance – co-operation projects and definition of references on the documented prevalence of diabetes

**DOI:** 10.25646/5988

**Published:** 2019-06-27

**Authors:** Christian Schmidt, Christin Heidemann, Alexander Rommel, Ralph Brinks, Heiner Claessen, Jochen Dreß, Bernd Hagen, Annika Hoyer, Gunter Laux, Johannes Pollmanns, Maximilian Präger, Julian Böhm, Saskia Drösler, Andrea Icks, Stephanie Kümmel, Christoph Kurz, Tatjana Kvitkina, Michael Laxy, Werner Maier, Maria Narres, Joachim Szecsenyi, Thaddäus Tönnies, Maria Weyermann, Rebecca Paprott, Lukas Reitzle, Jens Baumert, Eleni Patelakis, Thomas Ziese

**Affiliations:** 1 Robert Koch Institute, Berlin; 2 Institute for Health Services Research and Health Economics, German Diabetes Center (DDZ), Leibniz Institute for Diabetes Research at Heinrich Heine University Düsseldorf; 3 Institute for Health Services Research and Health Economics, Faculty of Medicine, Heinrich Heine University Düsseldorf; 4 German Center for Diabetes Research (DZD), Neuherberg; 5 German Diabetes Center, Leibniz Institute for Diabetes Research at Heinrich Heine University Düsseldorf, Institute for Biometry and Epidemiology; 6 Heidelberg University; 7 Institute for Applied Quality Improvement and Research in Health Care, Göttingen; 8 Central Research Institute of Ambulatory Health Care in Germany, Cologne; 9 Hochschule Niederrhein, University of Applied Sciences, Krefeld; 10 German Institute of Medical Documentation and Information, Cologne; 11 Helmholtz Zentrum München - German Research Center for Environmental Health, Institute of Health Economics and Health Care Management, Neuherberg

**Keywords:** DIABETES SURVEILLANCE, DIABETES MELLITUS, SECONDARY DATA, EPIDEMIOLOGY, PUBLIC HEALTH

## Abstract

In addition to the Robert Koch Institute’s health surveys, analyses of secondary data are essential to successfully developing a regular and comprehensive description of the progression of diabetes as part of the Robert Koch Institute’s diabetes surveillance. Mainly, this is due to the large sample size and the fact that secondary data are routinely collected, which allows for highly stratified analyses in short time intervals. The fragmented availability of data means that various sources of secondary data are required in order to provide data for the indicators in the four fields of action for diabetes surveillance. Thus, a milestone in the project was to check the suitability of different data sources for their usability and to carry out analyses. Against this backdrop, co-operation projects were specifically funded in the context of diabetes surveillance.

This article presents the results that were achieved in co-operation projects between 2016 and 2018 that focused on a range of topics: from evaluating the usability of secondary data to statistically modelling the development of epidemiological indices. Moreover, based on the data of the around 70 million people covered by statutory health insurance, an initial estimate was calculated for the documented prevalence of type 2 diabetes for the years 2010 and 2011. To comparably integrate these prevalences over the years in diabetes surveillance, a reference definition was established with external expertise.

## 1. Introduction

A pilot project to establish diabetes surveillance at the Robert Koch Institute (RKI) was launched in 2016. This step highlights diabetes mellitus’ great relevance to public health as a disease and cause of complications. Based on indicators agreed upon in consensus by experts, in future diabetes surveillance will report on diabetes-relevant developments as defined by its conceptual framework [1]. The presentation of results based on these indicators makes use of further data sources in addition to the data provided by the RKI in the context of health monitoring (primary data). The main objective of health monitoring is to provide representative information on the trends for the most important diseases, health behaviour and subjective assessments of the health of the population in Germany stratified by age, gender and socioeconomic status (see the article on Social inequality and diabetes mellitus in this issue)[2].

Conducting interview and examination surveys is time-consuming because the content must be coordinated with each other, and organisational planning and meticulous quality and data protection procedures also take time. Furthermore, as the objective is to collect information as efficiently as possible, the number of cases in primary studies is limited. To fully implement the objective of surveillance, i.e. provide in-depth stratification for specific indicators and at short time intervals, requires further sources of data. Reviewing potential sources of data, modes of accessing this data and adequate analyses were therefore key project tasks in the establishment of diabetes surveillance. To achieve these objectives, tenders for co-operation projects were organised in order to help define evaluation potentials, identify and close data gaps, and reveal options for data analysis for the entire duration of the project.

**Figure 1:**
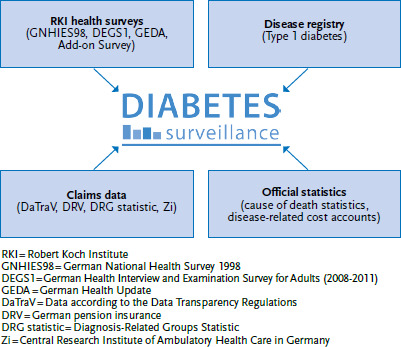
Data model of diabetes surveillance Own diagram

Out of the 40 indicators of diabetes surveillance 14 require only secondary data. A detailed description of indicators, their definition and the sources of data has been published on the RKI website [3]. 11 indicators that rely mainly on RKI health monitoring data also require secondary data. The diabetes surveillance data model (Figure 1) therefore includes RKI health monitoring, type 1 diabetes registry data (see the article on Type 1 diabetes in adults and type 2 diabetes in children and adolescents in this issue) as well as claims data of the statutory health insurance. This latter data is primarily collected for accounting and only secondarily used in scientific analyses, which is why such data has become known as secondary data [4]. Secondary data is process-produced data and therefore contains only limited information on socioeconomic status, subjective health, risk factors or undiagnosed diseases. The advantages of this data, however, are large samples and the availability of a constant inflow of new data.

Data from the social security system provides the core of secondary data, i.e. the data from the main providers of outpatient, inpatient and rehabilitative medicine: statutory health insurances (GKV) and the German pension insurance (DRV). Due to Germany’s regionally segregated health care structure, much of this routinely collected data is not centrally available, is processed by different institutions and therefore not always equally available to research [5]. Data on the provision of rehabilitation services through DRV for example are kept by the German pension insurance. Evaluating the use of medical rehabilitation services by diabetes patients is therefore only possible based on this data [6]. And GKV data is for example segregated by insurance providers and sector. While some health insurances do use the data of insured persons to calculate disease prevalences, the demographic and socioeconomic differences, as well as the differences in health risks between those insured by different insurers make comparisons difficult [5]. This makes it difficult to generalise, for example on the prevalence of diabetes among all people with statutory health insurance, and means prevalence can only be estimated based on assumptions [7].

**Info box 1:**

**Data by the Data Transparency Regulations (DaTraV)**

The data processing unit at the Institute of Medical Documentation and Information (DIMDI) can provide analyses based on the routine data of the around 70 million people covered by statutory health insurance (DaTraV data), which help plan, manage and assess the needs of the German health care system while ensuring the protection of the identity of ensured persons. DaTraV data can be evaluated across sectors of care and health insurance companies. It allows long-term assessments of disease progress for individual patients. As various publications have shown, using DaTraV data in the surveillance of noncommunicable diseases in particular principally makes sense. Yet there is room for improvements and an amendment to the data transparency regulations is planned. Changes will probably include

**►** Transferral of the diagnoses and medication data from the morbidity-oriented risk structure compensation scheme (Morbi-RSA), also of deceased individuals by the German Federal Insurance Office,
**►** reducing the delay in data provision from four to two years.

One aim is to reduce the current processing time for applications. Based on an external evaluation of the organisation, an increase of staff was applied for. Already, data on place of residence for 2009, 2010 and 2011 are available for analyses. From 2020, it will be possible to continuously analyse reporting years regionally by postal codes.

The Central Research Institute of Ambulatory Health Care (Zi) in Germany holds a complete set of outpatient claims data for all people covered by statutory health insurance. However, this data does not contain information on people covered by statutory health insurance with no outpatient consultations and totally lacks data on inpatient care [8]. Information on all people covered by statutory health insurance is contained in the data set held by the German Institute of Medical Documentation and Information (DIMDI) on the basis of Germany’s Data Transparency Regulations (DaTraV) [9]. However, this data too has its limitations. While it contains complete data on out- and inpatient diagnoses as well as prescribed medicines, it provides no information on inpatient or outpatient medical services. Furthermore, this data currently comes with a four-year delay and can so far only be evaluated regionally for individual reporting years. Moreover, neither the Zi nor the DIMDI data set provide information on people covered by private insurance. Info box 1 describes the data provided by DaTraV and its usability in surveillance systems, as well as an overview of planned reforms.

Beyond the problems posed by fragmented and incomplete data described above, the criteria to define diabetes in routine data (selection criteria) also vary. Differences in selection criteria, which can be justified based on content, produce different results and make comparability over time more difficult. Furthermore, particularly with diabetes, the differentiation between the different forms of the disease in analyses of secondary data is tied to a set of assumptions. Frequently one finds unspecific or aetiologically mutually exclusive diagnoses, such as a diagnosis of type 1 and type 2 diabetes simultaneously coded together [10]. A reference definition, which will be applied to DaTraV data, aims to increase the reliability, transparency and comparability of documented prevalence within the framework of diabetes surveillance. The definition of prevalence also provides the basis to determine further indicators such as those of mortality and incidence.

This article provides an overview of the results of the co-operation projects developed and co-operation partners found in the context of diabetes surveillance, who have all contributed significantly to the development of this project. We also present initial results on the prevalence of type 2 diabetes based on DaTraV data. The critical review of and experiences with this first set of results led to the definition of a frame of reference, which we also present here and which will provide the foundation for future diabetes surveillance reporting of documented prevalence and the calculation of further indicators.

## 2. Methodology

Since 2016, diabetes surveillance has published tender notices annually to promote co-operation projects. Suitable projects were selected based on specifically developed, standardised application and evaluation criteria, which were annually adapted in accordance with the stage of the project. The main criteria by which to evaluate the projects included a high public health relevance and easy integration within current surveillance, replicability of results, clarity of the method and feasibility of the project proposal within a one year time span. Developing indicators, sifting suitable sources of data and selecting co-operation projects were all done in parallel.

During a workshop in March 2017 [11], co-operation partners presented the identified data sources and exemplary analyses from projects implemented during the first funding years and discussed them with experts. During the workshop, DaTraV data and the possibilities to analyse this data using the data processing unit of DIMDI were presented and discussed. At the same time, we applied for an initial DaTraV output data set to calculate the prevalence of diabetes in 2010 and 2011. The results for type 2 diabetes were presented stratified by age group and gender and put in the context of current literature. Based on a critical contextualisation of results, in co-operation with the data processing unit and external expertise, we developed the reference definition presented here, which will serve as a basis to calculate documented prevalence and further indicators.

## 3. Results

In the following, the results of the cooperation projects from 2016 to 2018 will be presented first. Then the figures from the initial analysis of DaTraV data in the context of diabetes surveillance on the documented prevalence of type 2 diabetes during 2010 and 2011 will be presented and critically discussed.

### 3.1 Results of co-operation projects

Table 1 presents the authors of the co-operation projects, the indicators or contributions they worked on and the benefits these provide to diabetes surveillance.

#### 3.1.1 Time series on amputations and hospitalisation in patients with diabetes

**Figure 2:**
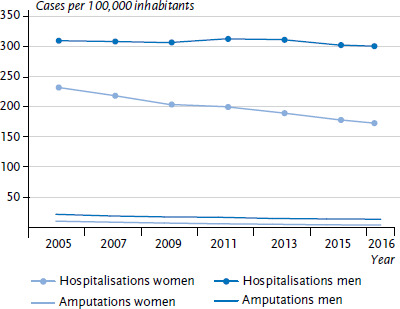
Hospitalisations and amputations over time (age standardised rates) for diabetes mellitus in Germany according to gender Source: Diagnosis-Related Groups Statistic (DRG statistic) 2005 to 2016

Hospitalisations due to lower limb amputations (major amputations) or other diabetes-related complications in diabetes patients are considered potentially preventable because diabetes can be controlled well with adequate structures for outpatient treatment. The Organisation for Economic Co-operation and Development (OECD) therefore uses hospitalisations as a population-related indicator that allows conclusions to be drawn on availability and quality of outpatient care [12]. These indicators are calculated for diabetes surveillance based on the Diagnosis-Related Groups Statistic (DRG statistic). 76,139 women with diabetes were hospitalised due to complications and 2,560 had amputations in Germany in 2016; for men, the figures were 108,386 hospitalisations due to complications and 5,402 amputations. After age standardisation using the German standard population in 2005, the number of hospital cases in the female population declined over time from 234 cases per 100,000 inhabitants in 2005 to 174 cases per 100,000 inhabitants in 2016. For men, the decrease was from 311 cases per 100,000 inhabitants in 2005 to 302 cases per 100,000 inhabitants in 2016 (Figure 2). The amputation rate for women during this same period dropped from 11.6 cases per 100,000 inhabitants to 5.4 cases per 100,000 inhabitants and from 23.0 cases per 100,000 inhabitants in 2005 to 14.9 cases per 100,000 inhabitants in 2016 for men. The development of rates could indicate an improved quality of outpatient care for diabetes patients over time or otherwise a greater adherence of patients to prescribed therapies [13].

Adjusted for age and gender, a regional focus reveals particularly high rates of hospitalisations due to complications as well as for amputations in the former East German federal states with the exception of Berlin [13]. The great influence of diabetes prevalence is an important factor to take into account here, i.e. regional differences in prevalence already explain the differences in rates of amputations and hospitalisations [14].

Integrating the OECD indicators in diabetes surveillance is easily feasible in terms of methodology, however, the changing definitions of indicators are challenging for comparisons over time. Over the course of the co-operation project, for example, the definition of amputations changed to exclude patients who had died in hospital. When interpreting indicators regarding their spatial distribution, predictors such as the regional differences in diabetes prevalence or socioeconomic factors must be taken into account.

#### 3.1.2 Potential uses for the Disease Management Programs

Disease Management Programs (DMP) are structured treatment programs for specific diseases that aim to improve treatment processes and the quality of care of chronically ill patients. A central feature of the disease management programs introduced for type 2 diabetes in 2003 and type 1 diabetes in 2006 is the quarterly or semi-annual documentation of standardised indicators by the participating doctor’s surgeries. Based on these indicators the DMP defines a set of quality objectives to describe the quality of treatment for patients in DMP cross-sectionally and longitudinally. Across Germany, 4.4 million patients are registered in the diabetes DMP. On average, inscribed patients have been attended to for 7.5 years (type 2 diabetes) or 7.1 years (type 1 diabetes) in the DMPs of the Association of Statutory Health Insurance Physicians Nordrhein (KV-Nordrhein) [15]. This provides a large data set that permits differentiated findings on quality of care by age or duration of illness.

The usability of DMP data is limited particularly due to the following factors:

1. As participation in DMPs is generally voluntary and programs are only open to patients in statutory health insurance who can still actively and independently handle their illness, selection effects are likely.
2. Documentation parameters and quality targets change over time, new indicators are created or indicators are replaced that have been used for many years. This creates gaps in surveillance over time.
3. Although the group of patients in the type 2 diabetes DMP is very large and probably very diverse regarding the duration of illness, there are no contractual bases to represent this diversity with regard to quality of care.

In spite of these limitations DMPs have provided a number of important results: diabetes DMPs reach a large proportion of diabetes patients. Over the course of time, the guideline-based treatment that diabetes patients receive has significantly improved. Continuous participation in DMPs increases the chances to achieve quality targets, and there is a clear decrease of severe complications for patients participating in type 2 DMPs. For diabetes surveillance, DMP data from North Rhine-Westphalia is presented to demonstrate the achievement of quality targets for both type 1 and type 2 diabetes. In future, the different frequencies of target achievement stratified by gender and age group over time will be reported and integrated into diabetes reporting.

#### 3.1.3 Routine data to measure quality: definitions and measurement

As part of this co-operation project, a complete data set for one statutory health insurance (AOK Baden-Württemberg) was used to analyse to what extent secondary data can contribute towards the overall project of establishing diabetes surveillance at the RKI.

To start, the aQua Institute for Applied Quality Improvement and Research in Health Care in Göttingen conducted a review of literature searching for type 2 diabetes indicators. In a first step it defined inclusion and exclusion criteria. Included were all indicators that can be applied to adult type 2 diabetes patients. Indicators of type 1 diabetes or gestational diabetes were excluded.

In November 2016, an expert panel took place in Göttingen. With external expertise, an indicator set was agreed in consensus. The experts evaluated all the indicators that had been found in the search in terms of their relevance for diabetes surveillance. The meeting offered room to further expand the indicator set. Consensus was found on 70 quality indicators.

47 of these 70 indicators, i.e. over two thirds of the set, can be calculated using secondary data. Importantly, however, this always requires evaluating the validity of the underlying secondary data. In addition, adequate procedures must ensure both internal and external validity, i.e. performing plausibility checks for results regarding the data itself (internal) as well as regarding other data sources (external) [17]. A report for the DIMDI on the access to and potential uses of data for care research [18] enumerates a number of advantages of GKV data has as well as its limitations. This report also considers other social insurance carriers, official statistics and federal health reporting, as well as private health insurance data.

Overall, the project made it very clear that secondary data, such as the data from AOK Baden-Württemberg used here, if specifically prepared and analysed, has the potential to close certain data gaps within a comprehensive diabetes surveillance approach. To reach a consensus on the set of 40 indicators, the indicators identified during the project were compared against the indicators found by the diabetes surveillance literature search. The project results led to the inclusion of these four additional indicators in the indicator set of diabetes surveillance: diabetic neuropathy, diabetic foot syndrome, renal replacement therapy and age at diagnosis.

#### 3.1.4 Epidemiologic parameters and projections for diabetes surveillance

The constant spread of diabetes poses considerable challenges to the healthcare system. Beyond taking stock of current case numbers, resource planning will require the most accurate prediction possible of future developments. Current estimates for Germany indicate that the number of diabetes patients is set to increase sharply in the future, these projections are however either based on figures for specific age ranges or solely on the data from particular statutory health insurances [19]. Due to the diversity of the demographic and socioeconomic characteristics of the groups insured by different health insurances, basing predictions on the data provided by individual insurances is problematic.

To better predict future figures of people with type 2 diabetes, this project applies the age- and gender-specific prevalence of type 2 diabetes from the year 2015 to the future age structure of the German population until 2040 as predicted by the Federal Statistical Office [20]. The assumed prevalence is thereby based on the data of all statutory health insurances in Germany, or DaTraV data [10].

Assuming that demographic ageing continues, yet that the age-specific prevalence of type 2 diabetes for women and men remains constant between 2015 and 2040, the number of people with type 2 diabetes will increase from 6.9 million in 2015 to 8.34 million in 2040, which would be a 21% increase. Predicting the future number of diabetes cases by using current age-specific prevalence is the simplest form to calculate a projection. Improved treatment of diabetes patients due to medical progress and the resulting longer average life expectancy will very likely lead to a rising age-specific prevalence, making the results presented here a conservative estimate. More realistic scenarios are obtained by modelling the interaction between incidence and mortality rates, which, when based on realistic assumptions of the trends until 2040, predict increases of over 50% [20].

#### 3.1.5 Using geocoding services to gain information on the obesogenicity of environments

A feasibility study tested the potential of data from online geocoding services to be used for the surveillance of environmental factors associated with type 2 diabetes. Using obesity as an example, initially the literature was searched for factors related to an obesogenic environment, i.e. an environment that is conducive to obesity [21-23]. Features of the environment that previous studies had associated positively with obesity were defined as obesogenic factors (for example fast food restaurants) and those defined as negative as protective factors (such as green areas). Subsequently, these factors were operationalised through expert interviews (n=4) based on the variables provided by the geocoding services Google Maps and OpenStreetMap. Using the statistics software R, new automated query scripts were developed to download and analyse relevant data in particular regarding the specific features of environments.

Site visits and an online search had the objective to validate the data from four pilot areas in Bavaria. This served to verify that the place and type of environmental features that had been detected were correct and whether there were additional relevant features that had been omitted from the identified data. The project showed that the demands for completeness of data, download capacity and the diversity of variables are relevant dimensions to select a particular geocoding service. Finally, for the city of Augsburg, kernel density estimations and heat maps were produced and cluster algorithms applied to describe the spatial distribution of variables. Cartographic analysis was then used to identify the areas with a high density of either obesogenic or protective environmental factors. Correspondingly, this study helped develop a suitable method to prepare and represent data from online geocoding services for the description of obesogenic environments suitable for diabetes surveillance. However, to assess the predictive power of this method for actual obesity and/or diabetes risks, the method will need to be tested in further surveys with population-based data.

#### 3.1.6 Indices relevant to public health and their projection relative to diabetes

The projection of diabetes-related years of life lost (YLL) and healthy life years (HLY) is based on prevalence, incidence and mortality rate ratio (MRR) data. Age- and gender-specific 2010 prevalences [10] were used to calculate YLL and HLY between 2015 and 2040 based on different scenarios of diabetes incidence and mortality.

As the long-term development of diabetes incidence in Germany is unknown, three hypothetical scenarios are currently being discussed: an unchanging incidence rate and an increase or a decrease in the incidence rate by 0.5%, respectively. Moreover, the advance of medicine will presumably lead to a greater decrease in the mortality of diabetes patients relative to people without diabetes, so the relative mortality risk is varied. We are therefore looking at scenarios with an annual 2% decrease in the mortality risk. YLL are calculated using a birth cohort framework, HLY by applying the Sullivan method [24].

During the period considered, the number of life years lost decrease for women and men. In 2040, people with diabetes will lose less life years than people without diabetes compared to 2015. This applies to all age groups. Women, in general, will lose less life years than men. Assuming an annually decreasing relative death risk between 2015 and 2040 would result in a relative decrease of YLL of up to 64% over the same period.

The model scenarios in their majority in principle indicate an increase of healthy life years (HLY) for the period considered across almost all age groups. One exception are the results for the over-80 age group. For example, an over-80-year-old man in 2040 can expect on average to live another 5 years without diabetes, whereas in 2015 it was 5.5 years. Our results are in line with the results of international studies on changes to diabetes-related morbidity [25].

#### 3.1.7 Renal replacement therapy in people with and without diabetes

Renal insufficiency belongs to the severe conditions diabetes patients may develop. At advanced stages this is treated with renal replacement therapy, which is associated with higher mortality and costs [26, 27]. For this reason, renal replacement therapy has been included as an indicator to diabetes surveillance, with the objective of analysing the disease burden over time [4]. However, there is so far no long-term data on whether the figures for renal replacement therapy (incidence) have decreased for people with and without diabetes in Germany.

So far, the incidence of renal replacement therapy has been analysed based on the data from doctors’ surgeries in North Rhine-Westphalia [26], as well as from the Gmünder Ersatzkasse (statutory health insurance data) of people with and without diabetes in the 2000s and provided results that can be compared well [28]: the age standardised incidence rate for the population with diabetes was between around 190 and 215 per 100,000 person-years, for the population without diabetes roughly between 30 and 40. Therefore, for diabetes patients the risk to have renal replacement therapy was around six to eight times higher. No significant trend over time was found. During the current project, the evaluation of data from doctors’ surgeries was expanded to cover the period between 2002 and 2016. The results are expected in 2019.

**Info box 2:**

**Reference definition to define documented prevalence of diabetes mellitus in the context of diabetes surveillance based on DaTraV data**

**Total diabetes**

*Denominator:* people insured for at least 360 days of one year with data on year of birth and gender, no insured residing abroad or those opting for reimbursement of costs according to section 13 (2) or section 53 (4) of Book 5 of the German Social Code (SGB V).

*Numerator:* people with at least two assured outpatient or at least one inpatient documented ICD-10 diagnoses of diabetes mellitus (E10.- to E14.-1)

**Type 1 diabetes**

*Denominator:* see above

*Numerator:* people with at least two assured outpatient documented ICD diagnoses E10.- or with an outpatient assured documented ICD diagnosis E10.- and at least one further ambulatory assured documented ICD diagnosis diabetes mellitus according to E12.- to E14.- or an inpatient documented ICD-10 diagnosis E10.-

*Excluded*: people with at least one ambulatory or inpatient documented ICD diagnosis E11.-

**Type 2 diabetes**

*Denominator:* see above

*Numerator:* people with at least two outpatient assured documented ICD diagnoses E11.- or with one outpatient assured documented ICD-10 diagnosis E11.- and at least one further outpatient assured diagnosis ICD-10 diabetes mellitus according to E12.- to E14.- or an inpatient documented ICD-10 diagnosis according to E11.-

*Excluded*: people with at least one outpatient assured diagnosis or inpatient documented ICD-10 diagnosis according to E10.-

**Other forms**

*Denominator: see above*

*Numerator*: people with at least two outpatient assured or at least one inpatient documented ICD-10 diagnosis in groups E10.- to E14.-

*Excluded*: people who were already assigned to type 1 or type 2 diabetes based on the algorithm mentioned above.

ICD-10 = International Statistical Classification of Diseases and Related Health Problems, 10th revision

E10 = Insulin-dependent diabetes mellitus (type 1 diabetes)

E11 = Non-insulin-dependent diabetes mellitus (type 2 diabetes)

E12 = Malnutrition-related diabetes mellitus

E13 = Other specified diabetes mellitus

E14 = Unspecified diabetes mellitus

An outpatient diagnosis can, depending on documentation, be a diagnosis that is suspected, related to a state following a certain illness (such as a heart attack), ruled out or assured. Only diagnoses classified as assured are used here to define documented prevalence.

**Validation data sets for total diabetes**

1. people who have been prescribed antidiabetic drugs without documented diabetes
2. people with only an inpatient secondary diagnosis

Moreover, there are plans to analyse the prevalence and incidence of renal replacement therapy in people with and without diabetes based on the data of several GKVs across Germany during the last decade. The project aims to analyse the possibilities to reliably describe renal replacement therapy through diagnoses based on the International Statistical Classification of Diseases and Related Health Problems (ICD) using health insurance data. The objective of this project is to evaluate the suitability of DaTraV data for this project, which as described above contains information on all statutory health insured. In a final step, a meeting of experts will discuss the comparability of different data sources (doctors’ surgeries, GKV and DaTraV data). One particular focus will be to discuss the possibilities to standardise the algorithms that apply to define renal replacement therapy and renal insufficiency in routine data.

### 3.2 Initial results for the documented prevalence of type 2 diabetes in DaTraV data

The first DaTraV application by diabetes surveillance referred to a comparison of the reporting years 2010 and 2011 for the documented prevalence of type 2 diabetes. Prevalent type 2 diabetes was defined as at least one assured documented out- or inpatient type 2 diabetes diagnosis coded according to the International Statistical Classification of Diseases and Related Health Problems, 10th revision (ICD-10: E11.-).

Together with experts from the diabetes surveillance scientific advisory board and based on the feedback from the data processing unit, the results of the query and of co-operation projects served as a starting point to define a reference analysis to determine documented prevalence (Info box 2). In future, this should provide robust results on the overall prevalence of diabetes as well as segregated by type 1, type 2 and other forms of diabetes mellitus for Germany and German federal states. The reference analysis will apply validation data sets. For one, the number of people who receive diabetes medication without having been diagnosed with diabetes will be determined. Furthermore, diabetics who according to the data only have a secondary inpatient diagnosis of diabetes will also be determined. This validation data set should help to better assess the consensually agreed definition.

**Figure 3:**
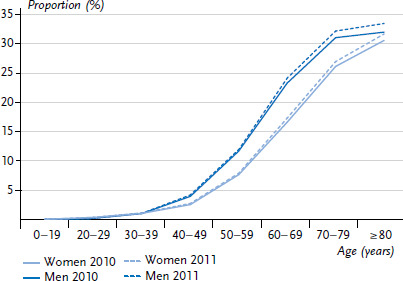
Comparison of the documented prevalence of type 2 diabetes mellitus for the years 2010 and 2011 according to gender Source: Data transparency ordinance data (DaTraV)

Irrespective of the results of the reference definition, the applied for results for the reporting years 2010 and 2011 show the DaTraV data potential for diabetes surveillance. Information on age, gender and diabetes diagnosis was analysed for a total of 66.2 million statutory health insured for the year 2010 and 66.4 million for 2011. The results in Figure 3 are stratified by gender and year for type 2 diabetes and evidence an increase in administrative prevalence. In women, documented prevalence increased from 7.7% (2010) to 8.1% (2011) and in men from 8.2% to 8.6%. Furthermore, the results confirm the known age-relatedness of type 2 diabetes, as for both genders an increase of prevalence with age is observed. The results resemble other analyses based on DaTraV data [10]. However, the age group over 80 is too large to describe the effect of decreasing prevalence among the very old aged over about 85 [8]. The definition of diabetes used for Figure 3, which bases itself on a definitive out- or inpatient diagnosis in the reporting year, differs from the generally used criterion of at least two quarterly periods. According to this criterion, a definitive outpatient diagnosis of diabetes must be coded during at least two quarters of one year to rule out documentation effects. The analysis moreover showed, that for numerous cases instead of specific diagnoses of either type 1 or type 2 diabetes, diagnoses were either unspecific or mutually exclusive. For these reasons, in co-operation with experts from epidemiology and care, the reference definition described below was developed to allow future description of the administrative prevalence of diabetes within the context of diabetes surveillance (Info box 2).

## 4. Discussion

Secondary data is an important element to determine indicators for diabetes surveillance. It can be used to prepare time series as a basis for numerous indicators.

In particular, DaTraV data is well suited for the purposes of surveillance, because it is a complete data set of all statutory health insured in Germany that is updated annually. The planned revision of DaTraV can overcome current limitations of this data set such as the transfer of diagnosis and medications data also for the year of leaving the GKV system and the current four-year delay. In addition, there is an objective to reduce the time required to process applications in particular by employing more staff. In future, this will ensure an even better depiction of regularly repeated observations of the disease burden at short intervals.

Meaningfully comparable analyses of secondary data require transparent and consensually agreed definitions of selection and applicability criteria. The presented reference definition to calculate the overall prevalence of diabetes, as well as differentiated by type 1, type 2 and other forms of diabetes based on DaTraV data is a step towards greater transparency. The developed definition thereby is not only important for comparisons of prevalence over time, but also serves as a reference for further indicators of diabetes surveillance. For example, the definition is also used to depict numerous diabetes complications, incidence and mortality based on DaTraV data.

Co-operation projects during the years 2016 to 2018 achieved a targeted transfer of results and methods in diabetes surveillance. In 2019, too, co-operation projects will be facilitated. Results from co-operation projects can be found on the diabetes surveillance website [29].

## Key statements

- 25 of 40 indicators of diabetes surveillance were either entirely or at least partially populated with secondary data.
- Over the course of the project, co-operation projects are promoted that specifically encourage the use of secondary sources of data.
- Co-operation project results indicate increasing case numbers by 2040 but also improved treatment for diabetes patients.
- Initial results, which are based on the data of all people covered by statutory health insurance, indicate an increase in documented prevalences of type 2 diabetes from 7.7% (2010) to 8.1% (2011) for women and 8.2% to 8.6% for men, respectively.
- A definition was developed that will serve as a basis for future calculations and enhance the reliability and comparability of results for documented prevalence in the context of diabetes surveillance.

## Figures and Tables

**Table 1:** Co-operation projects of diabetes surveillance, role within the project, authors and project description Own table

Co-operation project	Project year	Contribution	Use	Authors
Surveillance of ambulatory care-sensitive conditions in diabetes mellitus	2016	Amputations and hospitalisation (Section 3.1.1)	Regular presentation of indicators as a time series in surveillance	Johannes Pollmanns, Maria Weyermann, Saskia Drösler
Use of DMP documentation data for diabetes surveillance	No funding	All indicators of DMP quality assurance (Section 3.1.2)	Exclusive evaluation of DMP data for diabetes surveillance	Bernd Hagen
Measuring quality of care based on routine data	2016-2017	Feasibility study on the potential of GKV data (Section 3.1.3)	Comprehensive estimate as a basis for definitions and analyses based on secondary data	Gunter Laux, Joachim Szecsenyi, Stephanie Kümmel
Projections of prevalence and incidence of diabetes in Germany	2017	Prevalence prognosis models (Section 3.1.4)	Innovative epidemiological methods to model different scenarios for the development of number of cases	Ralph Brinks, Thaddäus Tönnies, Annika Hoyer
Co-operation with the data processing department to improve the use of DaTraV data in epidemiological research	No funding	Providing an overview of DaTraV data (Info box 1)	Reference evaluation with DaTraV data	Jochen Dreß
Feasibility study on the applicability of data on obesogenic environments in the surveillance of diabetes risk factors	2017	Obesity in tight-knit association with environmental factors (Section 3.1.5)	Analyses that make use of georeferential coding	Maximilian Präger, Christoph Kurz, Julian Böhm, Michael Laxy, Werner Maier
Updating of public health-relevant indices for diabetes surveillance and projections for the prevalence of diabetes and its limitations	2018	Disease burden figures (Section 3.1.6)	Use of biometric methods to estimate and provide prognoses for disease burden figures	Annika Hoyer, Thaddäus Tönnies, Ralph Brinks
Evaluation of St. Vincent targets based on diabetes mellitusrelated complications: terminal renal insufficiency in patients with or without diabetes	2018	Renal replacement therapy and renal insufficiency (Section 3.1.7)	Results from diverse data sources/development of definitions to use routine data	Heiner Claessen, Tatjana Kvitkina, Maria Narres, Andrea Icks

GKV = statutory health insurance, DaTraV = Data according to the data transparency regulations, DMP = disease management program(s)
